# Pre-Exposure to Cyclosporine A Primes *Paracoccidioides brasiliensis* to Evade Host Immunity

**DOI:** 10.1021/acsinfecdis.6c00285

**Published:** 2026-08-03

**Authors:** Filipe Nogueira Franco, Jean de Souza Lisboa Kozlowski, Nycolas Willian Preite, Coral Molist-Homs, Luiz Fernando de Oliveira, Ana Beatriz Furtado Rodrigues, João Henrique Dal Ponte Silva, Érica Borges Lima, Flávio Vieira Loures, Claudia Barbosa Ladeira de Campos

**Affiliations:** † Laboratory of Applied Immunology, Institute of Science and Technology, 28105 Federal University of São Paulo, Rua Talim, 330 - Vila Nair, São José dos Campos, São Paulo CEP 12231-280, Brazil; ‡ Laboratory of Biochemistry and Molecular Biology of Fungi (BioFun), Institute of Science and Technology, Federal University of São Paulo, Rua Talim, 330 - Vila Nair, São José dos Campos, São Paulo CEP 12231-280, Brazil

**Keywords:** paracoccidioidomycosis, cyclosporine A, calcineurin, natural product, adaptation

## Abstract

Paracoccidioidomycosis (PCM) is a systemic mycosis endemic to South America caused by thermodimorphic fungi of the genus *Paracoccidioides*, particularly *P. brasiliensis*. Calcineurin inhibition by cyclosporine A (CsA) perturbs fungal thermodimorphism and growth. Here, we asked whether pre-exposure of *P. brasiliensis* yeasts to CsA modulates host–pathogen interactions during infection. Mice infected with CsA-pre-exposed fungi exhibited significantly reduced levels of pro- and anti-inflammatory cytokines, along with decreased recruitment and activation of innate and adaptive immune cells at both time points. Although tissue inflammation and lesion areas were diminished, fungal burdens in lungs and liver were similar between groups. Importantly, yeasts recovered from lungs 72 h after infection with CsA-pretreated fungi displayed reduced *in vitro* colony growth, suggesting that prior CsA exposure induces a persistent slow-growth physiological state. This altered phenotype may impair effective immune recognition and inflammatory activation. As host immunosuppression was excluded in the experimental design, fungus-intrinsic phenotypes consistent with transient calcineurin pathway inhibition: altered growth/morphogenesis and putatively modified PAMP exposure that together blunt early immune activation. We interpret late-phase differences as downstream of these early pathway-dependent shifts rather than as heritable reprogramming. Overall, pre-exposure to CsA modulates *P. brasiliensis*–host interaction, dissociating tissue injury from fungal burden, highlights the role of calcineurin in fungal adaptation, and demonstrates how environmental exposure to bioactive compounds can influence fungal virulence and disease outcomes.

## Graphical abstract

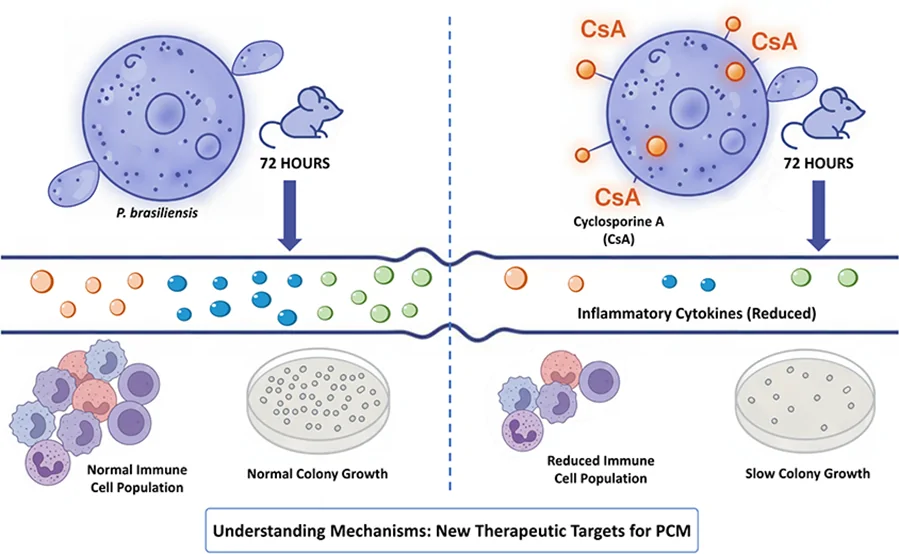

## Introduction

Fungal diseases represent a major and often underestimated public health challenge worldwide mainly due to an increasing problem of resistance to existing antifungals. It is estimated that more than one million deaths occur annually because of fungal diseases, making it a worrying issue.^1^ In this context, systemic mycoses endemic to specific geographic regions pose a particular challenge, as they are frequently underreported and poorly recognized outside their areas of endemicity. Among these diseases, Paracoccidioidomycosis (PCM) stands out as the most prevalent systemic mycosis in Latin America and the leading cause of death associated with fungal infections in Brazil, having the highest number of PCM cases (80%), followed by Colombia, Venezuela, Ecuador, and Argentina.^2^

Caused by fungi from the genus *Paracoccidioides*, mainly *P. brasiliensis* and *P. lutzii*, PCM presents two distinct clinical manifestations, the acute type, affecting mainly children under 15, more severe and immunologically distinct from the chronic form, predominantly in adult males, reflecting the complexity of host–pathogen interactions that shape disease outcomes.^3^ The acute form of the disease is characterized by the predominant activation of Th2/Th9 cells, and patients with the chronic form develop a mixed immune response with predominant differentiation of Th1/Th17/Th22 cells and high production of IL-17 and IL-22. Alternatively, individuals with asymptomatic infections without disease develop more polarized Th1 immunity.^4^ Infection is acquired mainly through inhalation of fungal propagules present in the environment, and epidemiological data consistently associate PCM with rural activities and soil exposure.^5^ In Brazil, it is found throughout the country and has been the main cause of death from systemic mycosis since ever.^6^

There are antifungal medications available against PCM that have different treatment protocols depending on the severity and age of the patient. However, long-term treatment can lead to sequelae due to chronic inflammatory processes and fibrosis, which can compromise the functions of affected organs, such as the lungs. Furthermore, persistent stimulation by the fungal antigen and activation of the immune system can lead to fibrosis through excessive deposition of extracellular matrix components and alterations in tissue healing, despite treatment.^7^ The need to find new strategies, medications, or a combination of both that can cure, control, reduce treatment’s time, or prevent new cases is fundamental to dealing with any mycosis. However, it is particularly difficult to define a crucial target to attack a pathogen when they so closely resemble their host. One of the most emblematic examples is perhaps calcineurin, the calcium- and calmodulin-dependent Ser/Thr phosphatase highly conserved throughout eukaryotes. In several fungi, calcineurin has been widely regarded as important for the regulation of many physiological processes, such as hyphal growth, ion homeostasis, thermotolerance, maintenance of the cell wall, resistance to antifungal drugs, and adaptation to stress, exhibiting usually fungistatic instead of fungicidal activity.^8,9^ The stress-adapting properties conferred by calcineurin are crucial for the virulence of fungal pathogens in plants and animals, which become avirulent when they lack calcineurin.^10^ Conversely, under nonstressful conditions, calcineurin knockout or its inhibition is mainly harmless for fungi.^11^ In *P. brasiliensis*, calcineurin activity is required for proper mycelium-to-yeast transition and sustained yeast proliferation at host temperature. Pharmacological inhibition of calcineurin by cyclosporine A (CsA) disrupts these processes, impairing fungal growth and morphogenesis, particularly under conditions that mimic the host environment.^9^

However, calcineurin is equally critical for mammalian physiology. In the host, calcineurin signaling is indispensable for T cell activation through the dephosphorylation of the nuclear factor of activated T cells (NFAT), leading to the transcription of cytokines essential for adaptive immune responses, such as IL-2, IL-4, IFN-γ, and TNF-α.^12,13^ For this reason, the calcineurin inhibitors cyclosporine A (CsA) and tacrolimus have been widely used as immunosuppressive drugs in organ transplantation and autoimmune diseases.^14^ Thus, this dual importance of calcineurin in both host and pathogen has long rendered it an unattractive target for antifungal therapy, as inhibition inevitably compromises host immunity.

Most experimental studies investigating the effects of CsA in fungal infections have therefore focused on drug administration to the host, often resulting in conflicting outcomes. While some reports describe reduced fungal burden and improved survival,^15,16^ others associate CsA treatment with increased susceptibility to infection due to immunosuppression.^17^ These discrepancies reflect the inherent difficulty in disentangling the direct effects of calcineurin inhibition on fungal physiology from its profound immunomodulation in the host.

An often-overlooked aspect of this interaction is that cyclosporine A and tacrolimus are not exclusively clinical or synthetic compounds, but natural products produced by soil-dwelling microorganisms that likely play ecological roles within their microbial communities. In soil ecosystems, secondary metabolites are known to modulate microbial interactions, competition for resources, niche partitioning, and stress adaptation.^18^ Yet, the ecological implications of calcineurin inhibition for pathogenic fungi inhabiting these environments remain largely unexplored. CsA was first isolated from *Tolypocladium inflatum* *Gams*, a soil fungus found in forests at Norway and Wisconsin, US, in 1970,^19^ whereas tacrolimus (FK506) was isolated from a soil bacterium in Japan in 1987, the *Streptomyces tsukubensis*.^20^ Since then, microorganisms that produce any of these compounds have been described elsewhere.^21^ It is worth noting that divergent microorganisms isolated from different geographic regions and environments, evolved the same, but distinct, mechanism to steadily block calcineurin activity. To do so, each compound must first bind to a specific class of prolyl-isomerases to then bind and inhibit calcineurin. While cyclosporine acts via the isomerase cyclophilin to complex with and then block calcineurin, tacrolimus does it via complexing with an FKBP (for FK506-binding protein) isomerase.^22^ For some human and plant fungal pathogens, certain cyclophilins are essential for virulence;^23^ for others, deleting one or the totality of cyclophilin genes may produce little or no impact on fungal viability.^24^

Although Paracoccidioides predominantly exists as mycelia/conidia in soil, climate stressors such as heat waves, El Niño–associated warming, and soil degradation can locally raise surface temperatures toward host-like conditions. Although environmental yeast forms of Paracoccidioides have not been demonstrated, localized warming associated with extreme climatic events could theoretically favor activation of the yeast developmental program within environmental microhabitats. This hypothesis remains speculative but provides a conceptual framework for investigating whether environmental exposure to calcineurin inhibitors may precondition fungi before host infection. In this context, environmental cyclosporine A (CsA)—a calcineurin-targeting metabolite produced by soil microorganisms—would not only interact with filamentous forms but could directly modulate cells engaging the yeast developmental program, the parasitic phase relevant to pathogenesis. Conversely, CsA present in soil before or during warming could inhibit or delay the M → Y transition. These climate-proximal scenarios motivate pathogen-focused experiments with yeast phase cells while acknowledging that environmental encounters with calcineurin inhibitors may precondition fungi prior to host entry.^25,26^

Most studies addressing calcineurin function in fungal virulence implicitly assume that inhibition of this pathway weakens the pathogen, rendering it more susceptible to immune control. However, this assumption may oversimplify a far more complex biological scenario. Fungi are remarkably adaptable organisms, capable of reprogramming their metabolism, growth dynamics, and stress responses in response to environmental challenges.^27^ Despite these considerations, little is known about how prior environmental exposure of *P. brasiliensis* to calcineurin inhibitors influences infection outcomes in immunocompetent hosts. Addressing this question requires an experimental design that isolates pathogen-intrinsic effects from host immunosuppression.

In this study, we examined the consequences of brief *in vitro* pre-exposure of *P. brasiliensis* yeast cells to cyclosporine A prior to experimental infection. After rigorous washout, we infected immunocompetent mice to assess whether CsA directly reprograms fungal physiology in a manner that affects host–pathogen interactions. We evaluated immune activation, inflammatory profiles, and disease outcomes at 72 h (acute) and 8 weeks (late), interpreting late-phase effects as downstream of early pathway-dependent shifts rather than as heritable reprogramming.

## Results

### Prior Exposure to CsA Induces Persistent Growth Impairment *In Vitro* without Loss of Viability

To evaluate the impact of cyclosporine A (CsA) pretreatment on fungal growth, *Paracoccidioides brasiliensis* yeast cells were exposed to CsA for 24 h, extensively washed, and subsequently cultured in CsA-free medium. Yeasts pretreated with CsA exhibited a marked reduction in their growth rate over 72 h compared to untreated controls, indicating impaired growth following transient exposure (Figure 1A). Growth impairment persisted for weeks after serial transfers into fresh medium (data not shown), consistent with a sustained fungistatic phenotype following washout. To assess whether this growth impairment persisted after infection, yeasts recovered from lungs 72 h postinfection were recultured *in vitro*. Fungi recovered from animals infected with CsA-pretreated yeasts displayed a delayed increase in colony numbers during the initial days of culture compared to fungi recovered from control animals (Figure 1B). From day 6 onward, colony counts converged between groups, indicating preserved viability despite delayed proliferation.

**Figure 1:**
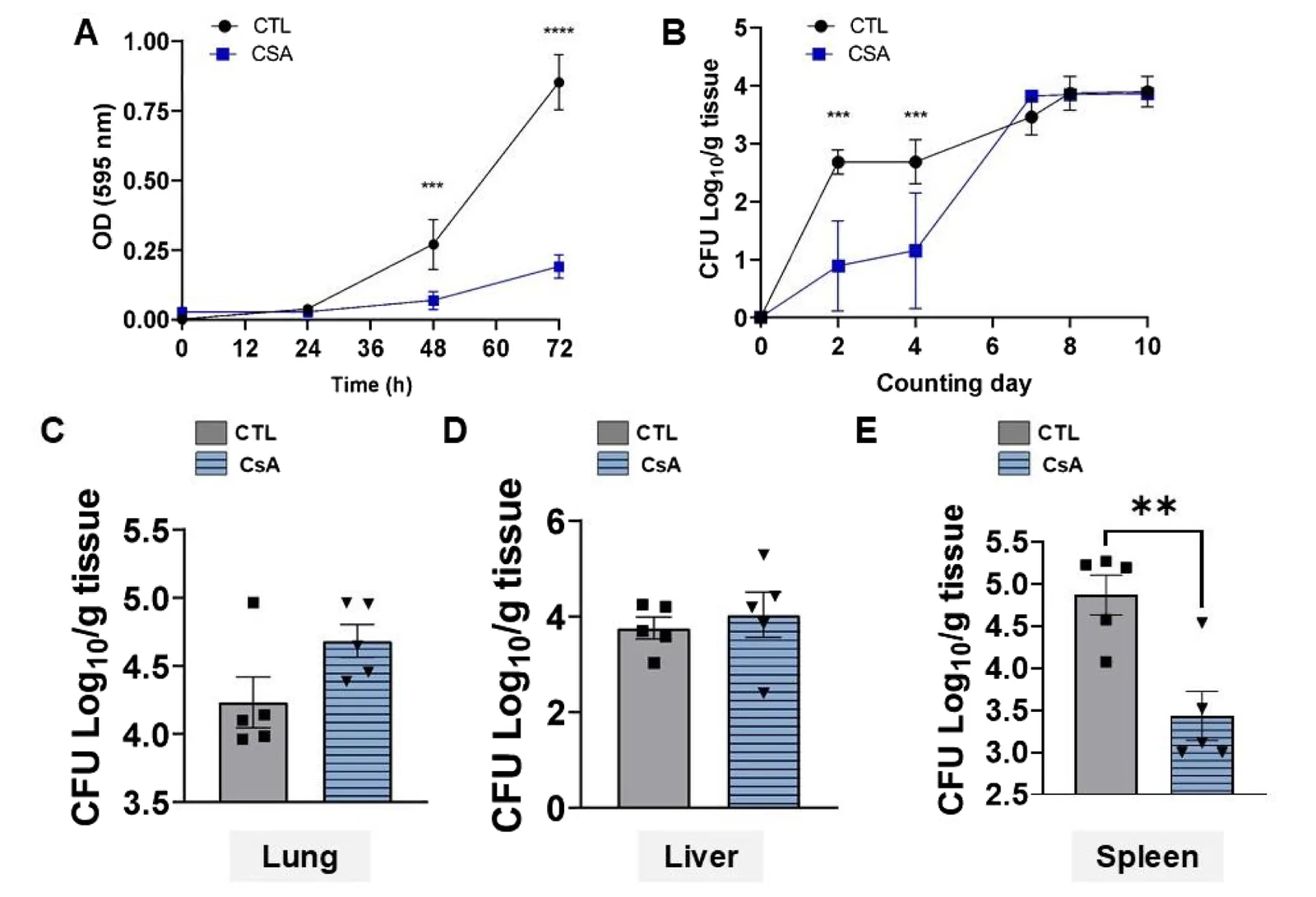
Brief pre-exposure to Cyclosporine A for 24 h induces a sustained fungistatic phenotype *in vitro* without reducing *in vivo* fungal burden. (A) Growth curve of yeasts cultured *in vitro* following treatment with 1 μg/mL of CsA for 24 h. (B-E) C57BL/6 mice were intratracheally infected with 10^6^ *P. brasiliensis* CTL yeasts or yeasts previously treated with CsA. After 72 h of infection, CFU was estimated in cultures of lung tissue from both groups (B). After 8 weeks of infection, CFU was estimated in lung (C), liver (D), and spleen (E). The data presented represent three experiments conducted with 4–5 mice per group. Comparisons between groups and means ± SD were analyzed by Student’s *t* test. Bars represent mean ± SD, based on four mice per sample. Values were considered significant when **p < 0.01, ***p < 0.001, and ****p < 0.0001.

During chronic infection (8 weeks), fungal burden in lungs and liver was similar between animals infected with control and CsA-pretreated yeasts (Figure 1C and 1D). In contrast, a reduced fungal burden was detected in the spleens of animals infected with CsA-pretreated fungi (Figure 1E). These data indicate that prior exposure to CsA delays fungal growth without abolishing the ability of *P. brasiliensis* to persist and disseminate *in vivo*.

### Reduced Tissue Inflammation Despite Preserved Fungal Burden during Chronic Infection

Histopathological analysis was performed to evaluate tissue inflammation during acute and chronic phases of infection. At 72 h postinfection, lung architecture and lesion area were comparable between animals infected with control or CsA-pretreated yeasts (Figure 2A). In contrast, during chronic infection (8 weeks), lungs of animals infected with CsA-pretreated fungi exhibited smaller granulomatous lesions and reduced inflammatory areas compared to controls (Figure 2B and 2C). A similar reduction in lesion area was observed in liver sections from animals infected with CsA-pretreated yeasts (Figure 2D and 2E). No marked histological differences were detected in spleens between groups (data not shown). These findings indicate that prior exposure of *P. brasiliensis* to CsA is associated with reduced tissue inflammation during chronic infection despite a comparable pulmonary burden.

**Figure 2:**
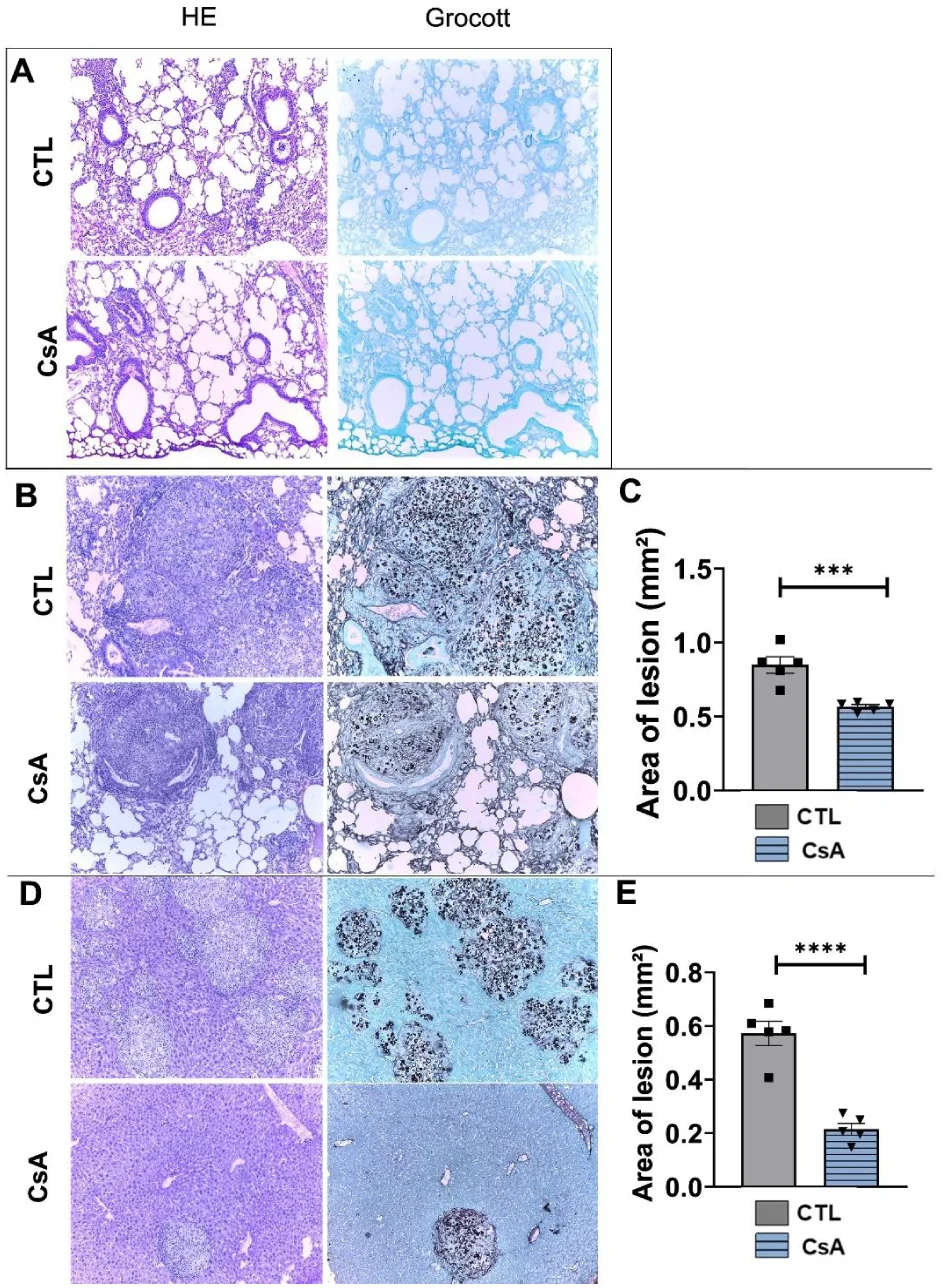
Histopathological analyses of animals infected with *P. brasiliensis* yeasts exposed to CsA show a reduction in lesion area, smaller granulomatous lesions, and decreased inflammatory cell infiltration. The histopathological examination was performed using hematoxylin–eosin and Grocott staining at 10× magnification. The total area of pulmonary and hepatic lesions was calculated in square micrometers (mm^2^), based on 5 microscopic fields per slide. (A) Lung tissue of the animals after 72 h of infection. (B-C) Lungs and (D-E) livers of the animals after 8 weeks of infection. Comparisons between groups and means ± SD were analyzed using Student’s *t* test. ***p < 0.001 and ****p < 0.0001.

### Attenuated Cytokine Production during Acute Infection in Mice Infected with CsA-Pretreated Yeasts

To assess early immune activation, cytokine levels were measured in lung homogenates 72 h after infection. Mice infected with CsA-pretreated yeasts exhibited reduced levels of multiple cytokines compared to animals infected with control fungi (Figure 3). Both pro-inflammatory (e.g., TNF-α, IL-6, IL-1β, IFN-γ) and regulatory/anti-inflammatory cytokines (e.g., IL-10) were significantly reduced in mice infected with CsA-pre-exposed yeasts at both time points. No consistent pattern corresponding to classical Th1, Th2, or Th17 immune polarization was observed at this time point. These data indicate that prior exposure of *P. brasiliensis* to CsA results in attenuated cytokine production during the acute phase of pulmonary infection. Conversely, the concentration of monocyte chemoattractant protein 1 (MCP-1) remained consistent in the lungs of both groups of infected mice, indicating that the recruitment of monocytes, memory T cells, and dendritic cells to sites of inflammation is likely maintained.

**Figure 3:**
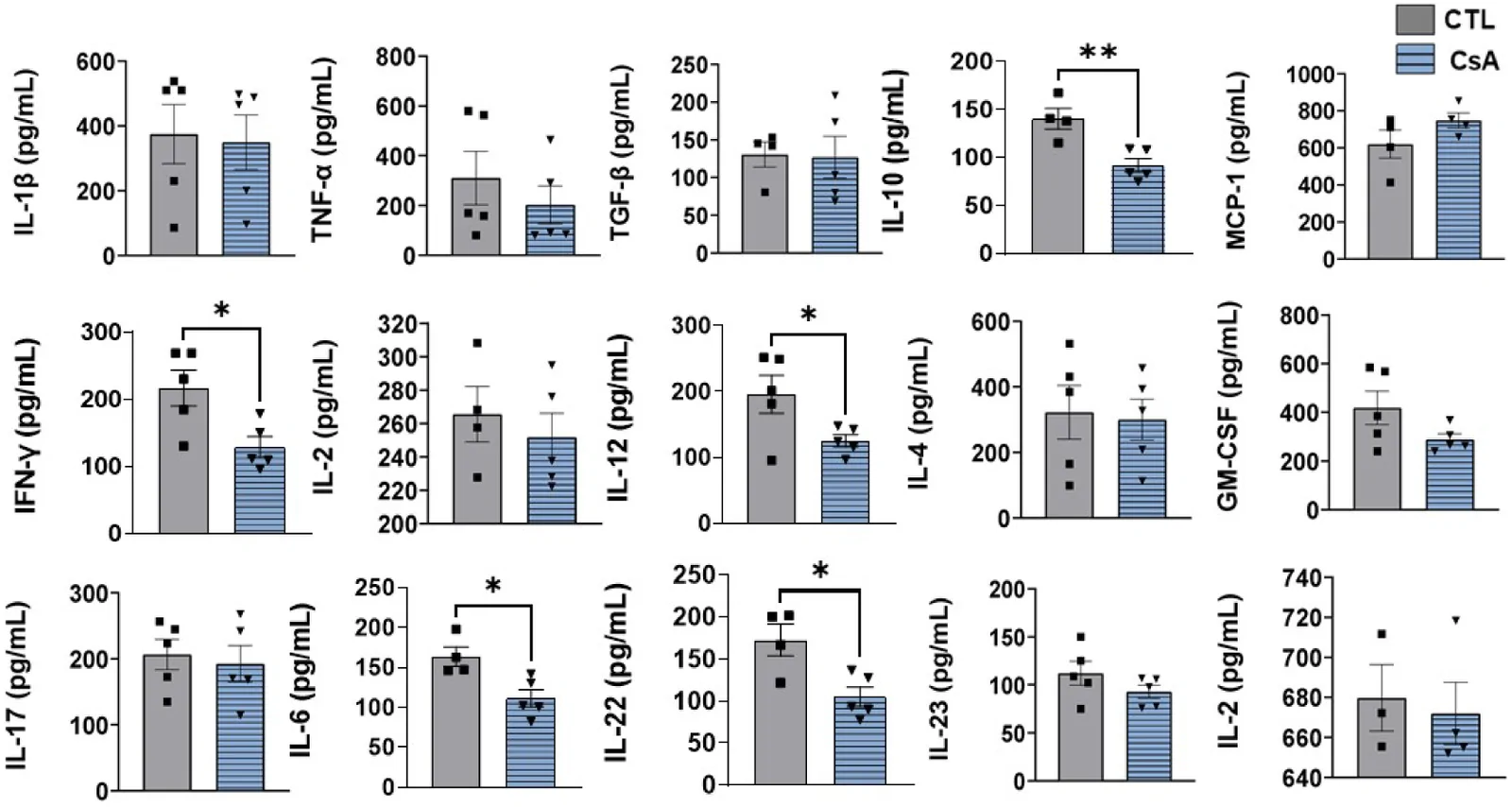
Cytokine analysis of animals infected with *P. brasiliensis*’ yeasts previously exposed to CsA shows an overall reduction in cytokine production. C57BL/6 mice were intratracheally infected with 10^6^ *P. brasiliensis* yeasts. After 72 h of infection, the animals were euthanized and the lungs were collected for cytokine quantification by ELISA. The data presented represent three experiments conducted with 4–5 mice each. Comparisons between groups and means ± SD were analyzed using Student’s *t* test. Values were considered significant when: *p < 0.05 and **p < 0.01.

### Attenuated Cytokine Production during Chronic Infection in Mice Infected with CsA-Pretreated Yeasts

Cytokine production was further evaluated during the chronic phase of infection, 8 weeks postinoculation, a time point at which fungal dissemination to secondary organs is established. Cytokine levels were quantified in homogenates from lungs, liver, and spleen. As shown in Table 1, lungs from mice infected with CsA-pretreated fungi exhibited a marked reduction in the production of nearly all cytokines analyzed, except for IL-23. In the liver, levels of TGF-β, IL-12, IL-4, IL-22, and IL-23 were also significantly reduced in this group. In contrast, in the spleen—the organ presenting the lowest fungal burden—only IL-1β and IL-10 levels were significantly decreased.

**Table 1:** Quantification of Cytokines in Lung, Liver, and Spleen Homogenates from Animals Infected with *P. brasiliensis* Yeasts Exposed to CsA^a^

**Cytokine**	**CTL**			**CsA**		
(pg/mL)	Lung	Liver	Spleen	Lung	Liver	Spleen
**IL-1β**	282.22 ± 53	783.33 ± 160	232.22 ± 80	172.77 ± 43*	663.88 ± 85	160.01 ± 19*
**TNF-α**	460.01 ± 31	827.78 ± 89	342.22 ± 78	356.11 ± 47**	700.03 ± 107	292.77 ± 83
**TGF-β**	455.01 ± 38	849.44 ± 88	358.33 ± 74	314.45 ± 49**	779.45 ± 66*	263.88 ± 66
**IL-10**	224.45 ± 57	210.03 ± 112	258.88 ± 109	173.88 ± 15*	182.25 ± 17	115.56 ± 19*
**MCP-1**	278.34 ± 37	788.89 ± 172	147.22 ± 91	214.45 ± 24**	579.45 ± 139	119.45 ± 32
**IFN-γ**	167.23 ± 34	629.45 ± 74	111.14 ± 93	122.23 ± 10*	726 ± 155	92.77 ± 37
**IL-2**	267.77 ± 16	577.22 ± 66	120.55 ± 24	191.94 ± 26**	485.55 ± 104	135.27 ± 27
**IL-12**	453.89 ± 183	964.11 ± 165	212.77 ± 46	305.55 ± 34*	846.66 ± 113*	307.78 ± 110
**IL-4**	308.89 ± 55	805.03 ± 132	278.05 ± 115	217.23 ± 47*	512.23 ± 85**	188.34 ± 64
**GM-CSF**	373.89 ± 38	811.12 ± 241	302.23 ± 72	237.78 ± 45**	762.78 ± 64	206.94 ± 42
**IL-17**	461.67 ± 84	849.45 ± 185	282.23 ± 128	303.34 ± 39**	756.67 ± 135	199.45 ± 54
**IL-6**	213.34 ± 29	844.16 ± 147	103.34 ± 40	127.23 ± 26*	873.05 ± 36	91.12 ± 19
**IL-22**	245.56 ± 58	793.34 ± 225	124.45 ± 81	132.23 ± 23**	536.67 ± 58*	88.34 ± 28
**IL-23**	416.12± 47	1115 ± 181	292.78 ± 169	300.56 ± 77	908.89 ± 75*	181.67 ± 71

a The results were considered significant using the Student’s *t* test, with *p < 0.05 and **p < 0.01.

Analysis of cytokine profiles associated with canonical Th1, Th2, and Th17 immune responses revealed that lungs from mice infected with CsA-pretreated yeasts did not display features consistent with any classical T helper polarization. Cytokines typically associated with Th1 responses, including IL-2, IL-12, IFN-γ, and TNF-α, Th2-associated cytokines IL-4 and IL-10, as well as cytokines involved in Th17 differentiation and maintenance, such as TGF-β, IL-17, IL-6, and IL-22, were broadly reduced in lung tissues at this stage of infection. Similarly, cytokine profiles detected in the liver and spleen did not correspond to any of the typical Th-associated immune response patterns (Table 1).

### CsA Pretreatment Reduces Early Recruitment of Innate Immune Cells to the Lungs

To determine whether prior exposure of *P. brasiliensis* to CsA affected early immune cell recruitment, lung-infiltrating leukocytes were analyzed by flow cytometry 72 h after infection. Mice infected with CsA-pretreated yeasts exhibited a significantly lower frequency of total leukocytes (CD45^+^) in lung homogenates compared to animals infected with control yeasts (Figure 4). This reduction was accompanied by decreased frequencies of total myeloid cells (CD45^+^CD11b^+^), macrophages (Mφ), dendritic cells, and neutrophils. Analysis of absolute cell numbers revealed a similar trend. Mice infected with CsA-pretreated fungi showed reduced numbers of dendritic cells and neutrophils in the lungs, whereas macrophage numbers were modestly reduced or unchanged depending on the experiment (Figure 4). Thus, despite MCP-1 presence in tissue lesions, the prior exposure of *P. brasiliensis* to CsA impairs early recruitment of innate immune cells to the lungs during acute infection.

**Figure 4:**
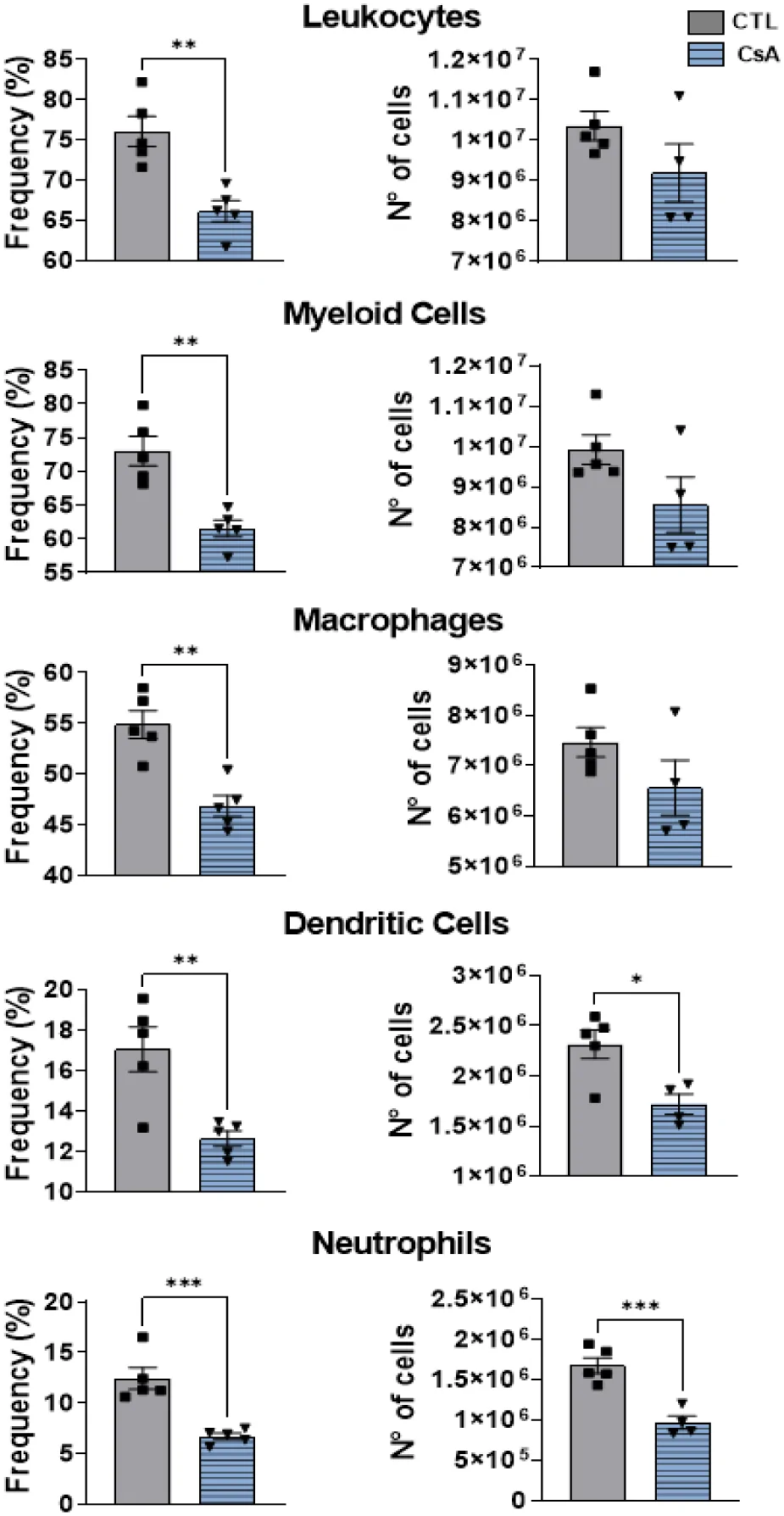
Frequency and number of leukocytes, myeloid cells, macrophages, dendritic cells, and neutrophils were lower in the lung-infiltrating leukocytes from mice infected with fungi pretreated with CsA. C57BL/6 mice were intratracheally infected with 10^6^ *P. brasiliensis* yeasts previously treated or not with CsA. After 72 h of infection, the animals were euthanized and the lungs were collected and processed to release the migrated cells. Dead cells were excluded from the analysis using a Live/Dead **dye**. Leukocyte populations were identified using the following phenotypes: CD45^+^ (only leukocytes); CD45^+^CD11b^+^ for myeloid cells; CD45^+^CD11b^+^F4/80^+^ for macrophages; CD45^+^CD11b^+^CD11c^+^F4/80^–^ for dendritic cells; and CD45^+^CD11b^+^Ly6G^high^ for neutrophils. Data analysis was done by FlowJo software, and comparisons between groups, averages ± the standard deviation obtained were analyzed by Student’s *t* test. The bars represent averages ± SD of 4–5 mice/group. *p < 0.05, **p < 0.01 and ***p < 0.001 using the Student’s *t* test.

### Reduced Activation Phenotype of Macrophages and Dendritic Cells in Mice Infected with CsA-Pretreated Yeasts

Because macrophages and dendritic cells play a central role in antigen presentation during pulmonary Paracoccidioidomycosis, we next evaluated the expression of activation and costimulatory molecules on these populations in lung infiltrates 72 h postinfection. Flow cytometric analysis revealed that mice infected with CsA-pretreated yeasts displayed lower frequencies of macrophages and dendritic cells expressing CD80 and CD40 compared to control animals (Figure 5). In addition, a reduced frequency of CD86-expressing dendritic cells and diminished expression of MHC-II on both macrophages and dendritic cells were observed in lungs of mice infected with CsA-pretreated fungi. Absolute numbers of activated antigen-presenting cells followed the same pattern, with fewer CD40^+^ and CD80^+^ cells detected in this group (Figure 5). These results demonstrate that prior exposure of *P. brasiliensis* to CsA is associated with a reduction of both recruitment and activation phenotype of lung antigen-presenting cells during early infection.

**Figure 5:**
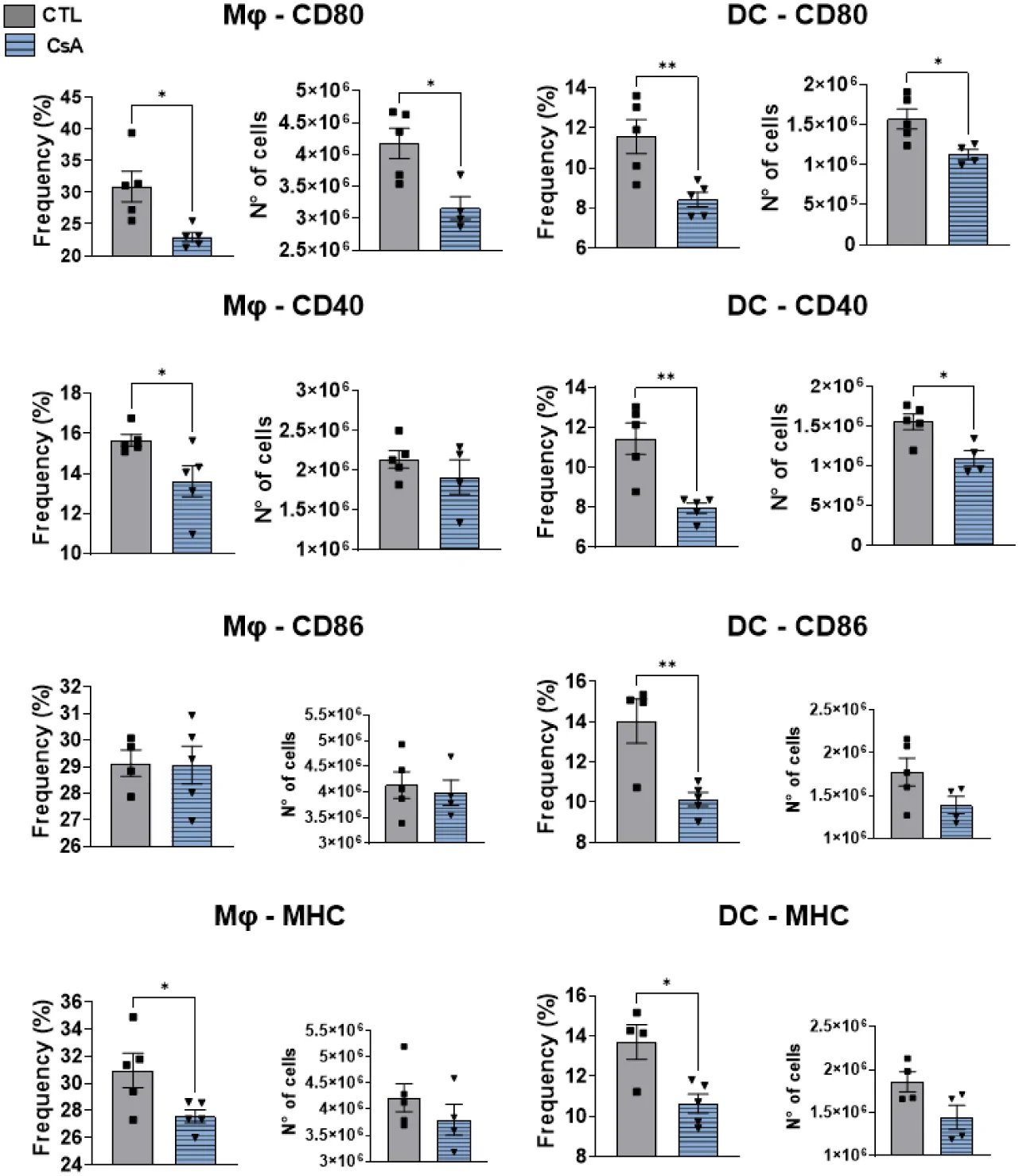
Frequencies and numbers of macrophages and dendritic cells were lower in the lung-infiltrating leukocytes from mice infected with fungi pretreated with CsA. C57BL/6 mice were infected intratracheally with 10^6^ *P. brasiliensis* yeasts previously exposed or not with CsA **and washed**. After 72 h of infection, the animals were euthanized, and the lungs were collected, processed to release the migrated cells, and labeled for specific markers of cell activation. Macrophages expressing activation molecules (CD40, CD80, CD86, and MHC-II) were defined as CD45^+^CD11b^+^CD11c^+^F4/80^+^ cells. Dendritic cells expressing the same activation molecules were identified as CD45^+^CD11b^+^CD11c^+^F4/80^–^ cells. Data analysis was done by FlowJo software; and comparisons between groups, averages ± the standard deviation obtained were analyzed by Student’s *t* test. The bars represent averages ± SD of 4–5 mice/group. *p < 0.05 and **p < 0.01 using the Student’s *t* test.

### Attenuated Recruitment and Activation of T Lymphocyte Subsets during Acute Infection

To assess whether alterations in innate immune activation were accompanied by changes in lymphocyte responses, CD4 and CD8 T cell populations were analyzed in lung infiltrates 72 h postinfection. Mice infected with CsA-pretreated yeasts exhibited a significantly reduced frequency and number of total CD4^+^ T lymphocytes compared to control animals (Figure 6). Further phenotypic analysis revealed lower frequencies and numbers of activated CD4^+^CD25^+^ T cells, as well as reduced representation of Th1 (IFN-γ^+^), Th2 (IL-4^+^), Th17 (IL-17^+^), and regulatory T cell (Foxp3^+^) subsets in the lungs of mice infected with CsA-pretreated fungi (Figure 6). In contrast, the total frequency of CD8+ T cells was largely unchanged; however, activated CD8^+^CD69^+^ cells and Tc1, Tc2, and Tc17 sub**sets** were reduced in this group (Figure 6). Collectively, these data show that prior exposure of *P. brasiliensis* to CsA results in diminished recruitment and activation of multiple innate and adaptive immune cell populations during the acute phase of pulmonary infection.

**Figure 6:**
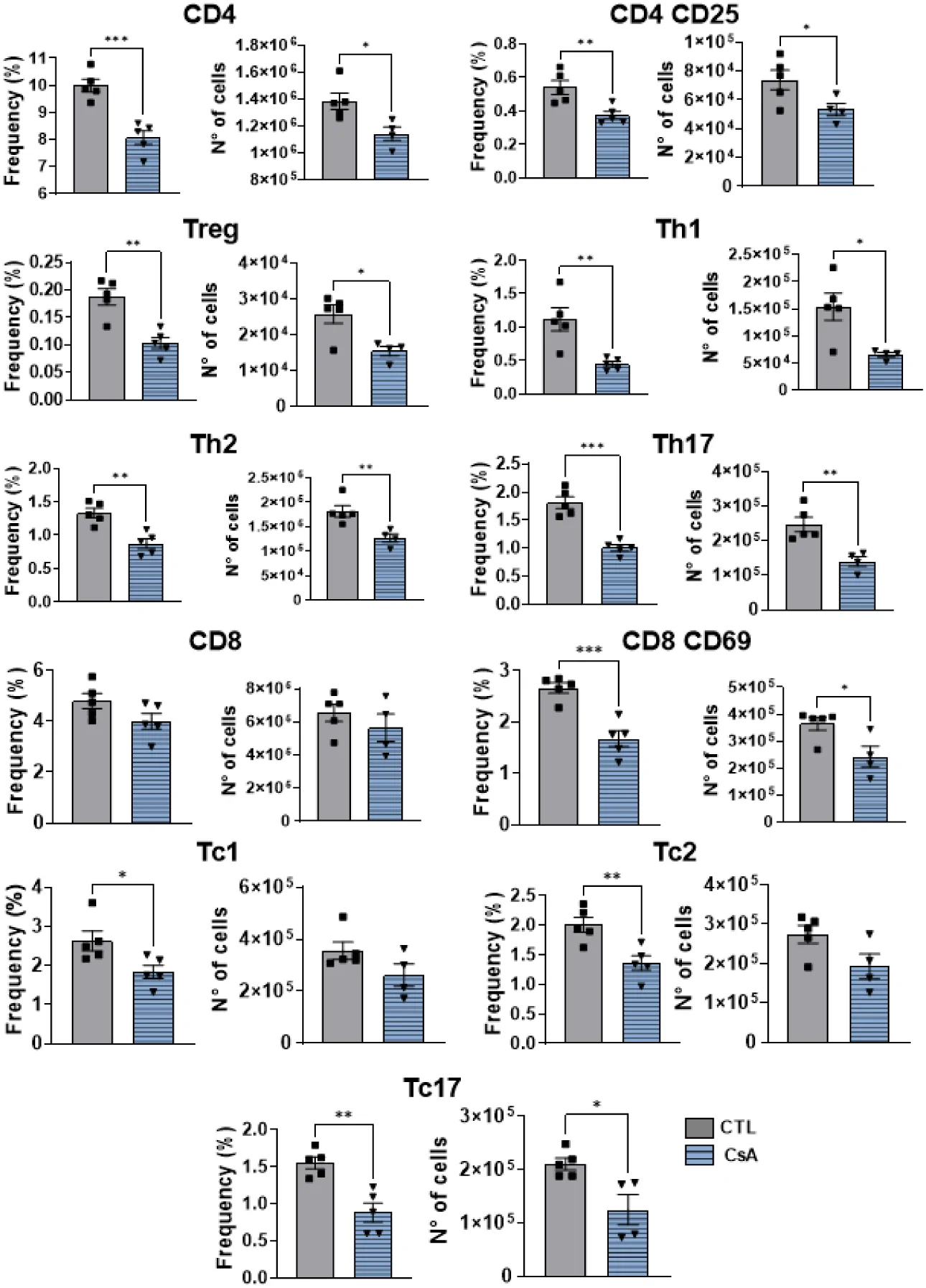
Frequency and number of CD4 and CD8 T lymphocytes were lower in the lung-infiltrating leukocytes from mice infected with fungi pretreated with CsA. C57BL/6 mice were infected intratracheally with 10^6^ *P. brasiliensis* yeasts exposed or not with CsA. After 72 h of infection, the animals were euthanized, and the lungs were collected, processed to release the migrated cells, and labeled for typical markers for T cells. Lymphocyte populations were identified as CD4^+^/CD8^+^ and CD4^+^CD25^+^/CD8^+^CD69^+^ cells. Intracellular staining was performed to identify Th1 (CD4^+^IFN-γ^+^), Th2 (CD4^+^IL-4^+^), Th17 (CD4^+^IL-17^+^), Tregs (CD4^+^CD25^+^Foxp3^+^), Tc1 (CD4^+^IFN-γ^+^), Tc2 (CD4^+^IL-4^+^), and Tc17 (CD4^+^IL-17^+^) cells. Data analysis was done by FlowJo software, and comparisons between groups, averages ± the standard deviation obtained were analyzed by Student’s *t* test. The bars represent averages ± SD of 4–5 mice/group. *p < 0.05, **p < 0.01 and ***p < 0.001 using the Student’s *t* test.

## Discussion

This study investigates calcineurin inhibition through both experimental methods and theoretical analysis, considering the impact of ecological and environmental histories of pathogens on immune responses and disease outcomes. We emphasize pathogen-intrinsic consequences of transient calcineurin pathway inhibition, under conditions explicitly excluding host immunosuppression, highlighting that microbiology and host–pathogen biology can benefit from cross-disciplinary perspectives that connect environmental chemistry to infectious outcomes.^28^ To probe specificity, we evaluated alisporivir (Debio 025), a nonimmunosuppressive CsA analog that inhibits cyclophilins but does not inhibit calcineurin. In our *in vitro* assays, alisporivir did not impair yeast growth under the conditions tested, whereas CsA altered growth/morphology and proliferation. Taken together, these observations support that the effects we report are attributable to calcineurin inhibition rather than a cyclophilin-only effect.

Cyclosporine A is classically associated with immunosuppression in mammals through the inhibition of NFAT-dependent transcription required for T cell activation.^29,30^ This creates an interpretive challenge in infection models where CsA is administered to the host, as outcomes reflect inseparable effects on host immunity and pathogen physiology. Here, mice were not immunosuppressed since CsA exposure was restricted to the fungus *in vitro*, followed by extensive **washout** prior to infection, thereby excluding pharmacologically relevant host exposure. Given that murine immunosuppression protocols require repeated CsA administration over extended periods at substantially higher doses,^31,32^ host immunosuppression is an unlikely explanation for the phenotypes observed. Instead, the data support a model in which transient calcineurin inhibition modulates fungal growth/morphogenesis and early host interaction.

In our previous study, pharmacologic inhibition of calcineurin with cyclosporine A (CsA) altered filamentous-phase growth and hyphal morphology at 25 °C, compromised the mycelium-to-yeast transition at 37 °C, and perturbed the yeast morphology under host-like temperature conditions.^9^ In this work, brief exposure of *P. brasiliensis* yeasts to CsA imposed durable effects on fungal growth dynamics after washout, evidenced by minimal 72 h growth of the same inoculum in parallel F12 cultures and delayed proliferation of yeasts recovered from lungs at 72 h. Notably, despite morphological alteration of mycelia and yeasts under CsA treatment and this growth impairment of the yeasts recovered from lungs, CsA-pretreated fungi retained the ability to establish infection and disseminate *in vivo*, achieving fungal burdens comparable to controls during chronic infection. Also, animals infected with CsA-pretreated yeasts exhibited reduced inflammatory lesions and milder tissue pathology. This dissociation between persistence and host damage indicates preserved pathogenicity with reduced virulence. A plausible explanation is that early slowing of fungal replication and/or altered PAMP exposure/organization raises the thresholds for innate sensing, limiting inflammatory amplification while allowing persistence and dissemination with reduced tissue injury. In this framework, the host interaction phenotypes we observe after CsA pre-exposure are consistent with modified PAMP presentation and/or growth phase kinetics rather than loss of viability. While we did not directly quantify cell wall components or receptor engagement here (e.g., dectin-1 Fc binding, WGA/ConA staining, aniline blue assays), our findings align with a calcineurin-dependent remodeling of cell wall organization that can shape innate recognition during infection.^33,34^ Therefore, the observed phenotypes were: (i) calcineurin-dependent wall/morphology remodeling may change PAMP exposure; and (ii) calcineurin-induced slowed growth kinetics can reduce innate sensing thresholds or alter timing of recognition. We therefore interpret the phenotype as reduced virulence (extent of host damage) without loss of pathogenicity (capacity to establish infection). Slower growth rates may not necessarily reflect reduced fitness but could instead represent an adaptive strategy that minimizes immune detection and inflammatory activation.^9,35^

From this perspective, the distinction between pathogenicity and virulence becomes particularly relevant. A pathogen that proliferates slowly yet attenuates immune activation via altered wall organization and growth phase kinetics may retain pathogenicity while exhibiting reduced virulence, that could be advantageous for its long-term survival. However, this does not mean, in any way, that decreased virulence is related to a greater competence of the immune system to recognize and eliminate the pathogen. The ability of CsA-pre-exposed fungi to regain growth *in vivo* also highlights the permissive nature of the host environment, which provides nutrients or cues absent from defined media. These findings argue against a potential transient toxicity of CsA to pretreated fungi and instead indicate a transient, pathway-dependent state that blunts early immune activation without compromising viability.

Reduced cytokine production was observed during both acute and chronic phases of infection across multiple organs, indicating an overall attenuation of the immune response. This pattern was reflected at the cellular level, with decreased recruitment and activation of leukocytes, myeloid cells, macrophages, dendritic cells, and CD4 T cell subsets early after infection (72 h). We also observed a reduced expression of MHC-II and key costimulatory molecules (CD40, CD80, CD86) on macrophages and dendritic cells- features consistent with impaired antigen presentation and blunted T cell priming. We did not directly quantify β-glucan/chitin/mannan exposure or PRR engagement; thus, we frame altered PAMP presentation as a plausible, testable mechanism rather than a demonstrated cause. In the absence of omics data and instead attributing acute effects to ongoing pathway inhibition after washout, late-phase differences were viewed as downstream of altered early colonization and immune set points.

PCM has traditionally been classified into acute/subacute and chronic forms, with additional nonclassical presentations described in immunocompromised patients, such as those coinfected with HIV.^36^ Our findings extend this conceptual framework by demonstrating that fungal preconditioning prior to infection can generate a distinct outcome profile in immunocompetent hosts: immune hypoactivation, impaired antigen presentation, and reduced tissue inflammation despite a comparable lung burden. This phenotype arises from fungus-intrinsic calcineurin inhibition at the time of host entry, not from host-directed immunosuppression.

Cyclosporine A is a natural product produced by soil microorganisms, and related compounds may plausibly occur in environments inhabited by *Paracoccidioides* sp.^37^ Our results provide experimental support for the idea that environmental exposure to calcineurin targeting metabolites can precondition fungal pathogens, shape host–pathogen interactions, and influence disease outcomes. Considering ecosystem degradation, global warming, soil desertification, and antifungal use in agriculture, heat-driven yeast-like states and co-occurrence with CsA-producing microbes may have unpredictable consequences for PCM and other dimorphic mycoses.^38^ Paracoccidioides predominantly exists in the environment as mycelia and conidia,^39^ and there is currently no direct evidence demonstrating the presence of naturally occurring yeast or yeast-like cells in soil or other environmental niches. We acknowledge that natural infection is initiated by inhalation of conidia rather than environmental yeast cells. Therefore, the ecological scenario proposed here should be viewed as a conceptual hypothesis rather than a demonstrated environmental pathway. Nevertheless, because Paracoccidioides is a thermodimorphic fungus, transient warming associated with extreme climatic events could theoretically promote localized activation of the yeast developmental program within environmental microhabitats. This possibility remains speculative, requires experimental validation, and warrants future investigation. If such conditions occur, environmental exposure to cyclosporine A (CsA) could potentially influence cells entering the yeast developmental program, thereby affecting cell wall organization and subsequent host–pathogen interactions. Our experimental design instead focused on determining whether transient calcineurin inhibition of the pathogenic yeast phase is sufficient to modify subsequent host–pathogen interactions.

Recently, there have been proposals to use cyclosporine as an insecticide in agriculture because it suppresses the immune systems of insect pests.^40,41^ However, applying cyclosporine to crops should be avoided since it might reduce the immune defenses of humans and animals, and even turn normally harmless fungi into pathogens.^42^ While our data focus on pathogen-intrinsic effects in a controlled model, they underscore a broader ecological link between environmental chemistry and virulence modulation. This ecological perspective represents a major conceptual advance in understanding how environmental factors may influence the emergence of fungal diseases. Also, future studies should evaluate whether other immunosuppressive agents similarly modulate fungal virulence and host immunity. In addition, it will be important to determine whether transient calcineurin inhibition also alters susceptibility to cell wall-targeting antifungal agents. Given the established role of calcineurin in maintaining cell wall integrity and coordinating stress responses, future studies should investigate whether the phenotypic changes induced by transient CsA exposure influence antifungal susceptibility, thereby expanding our understanding of how environmental exposure to immunomodulatory compounds may shape fungal pathogenicity and therapeutic responsiveness.

## Conclusions

Pre-exposure of *Paracoccidioides brasiliensis* yeasts to cyclosporine A, followed by washout, yields fungus-intrinsic phenotypes consistent with transient calcineurin inhibition: sustained fungistatic behavior after washout, attenuated immune activation, and reduced tissue damage, despite comparable pulmonary fungal burden. We interpret late-phase differences as downstream of early pathway-dependent shifts in colonization and immune set points, rather than as heritable reprogramming. Beyond calcineurin biology, these findings highlight how environmental bioactive compounds can precondition dimorphic fungi and shape disease outcomes, supporting a distinct nonclassical outcome profile of PCM driven by pathogen preconditioning.

## Methods

### Culture of *P. brasiliensis* and Cell Density Determination

Stocks of *P. brasiliensis* strain 18 (Pb18) yeasts were maintained at 4 °C in solid YPD medium and renewed monthly. Before experiments, cells were reactivated and subsequently inoculated into a preinoculum culture, both performed in Ham’s F-12 medium supplemented with 2% glucose and incubated at 37 °C with shaking at 130 rpm, for 3 days each. The concentration of yeasts in the preinoculum was adjusted to 1 × 10^6^ cells·mL^–1^ using a Neubauer chamber (or OD_595_ ≈ 0.01) for further *in vitro* culture in fresh Ham’s F-12 with 2% glucose, in the absence or presence of 1 μg·mL^–1^ cyclosporine A (CsA) at 37 °C and 130 rpm. After 24 h, CsA was washed out by five sequential washes with sterile PBS in 15 mL tubes at 3,000 × g for 5 min. Cells were then disaggregated by passage through syringe needles of decreasing gauge (18G 1 1/2, 19G 1 1/4, and 23G 1), 10 times each, and adjusted to 2 × 10^7^ cells·mL^–1^ using a Neubauer chamber. Yeast viability was assessed immediately after CsA exposure and immediately prior to inoculation using trypan blue (Live/Dead dye) exclusion under light microscopy; only unstained (membrane-intact) cells were counted as viable. These cell suspensions were used for both murine infection (50 μL or 10^6^ cells per animal) and parallel *in vitro* growth in Ham’s F-12 with 2% glucose. Samples were collected daily, fixed in 2% paraformaldehyde, and optical density (OD) at 595 nm was used to measure cell growth.

### Animals and Infection

Male wild-type C57BL/6 mice (8–12 weeks old) were used in this study. Animals were obtained as specific pathogen-free from CEDEME-UNIFESP and maintained at the NB2 Animal Facility, UNIFESP. All procedures were approved by the Institutional Animal Ethics Committee (No. 9124020224).

Mice were randomly assigned to experimental groups prior to infection. All animals were age-matched (8–12 weeks old), which reduces variability in body size and physiological status at the start of the experiment. Mice had free access to food and water and were anesthetized with ketamine (90 mg/kg) and xylazine (10 mg/kg) prior to infection. Intratracheal inoculation was performed with 1 × 10^6^ yeasts in 50 μL PBS (from 2 × 10^7^ cells/mL cell suspensions) to ensure direct lung delivery. Four groups were evaluated: untreated (CTL) and cyclosporine A-pre-exposed (CsA) yeasts in both acute (72 h) and chronic (8 weeks) infection models. These two time points were selected based on our previous characterization of the murine PCM model. First, the 72 h time point was used to evaluate the early stage of pulmonary infection, mainly characterized by innate immune responses and initial cytokine production following fungal establishment in the lungs. Second, on the 8 week time point, infection is chronically established with persistent fungal burden, organized granulomatous lesions, and T cell exhaustion.^43^

### CFU Assays and Histological Analysis

The lungs, liver, and spleen from each infected animal were collected individually and placed in 5 mL of sterile phosphate-buffered saline (PBS). Each tissue was mechanically dissociated using a Potter–Elvehjem homogenizer until a homogeneous suspension was obtained. The homogenates were transferred to 15 mL centrifuge tubes, properly labeled according to organ and experimental group. Samples were then centrifuged at 3,000 × g for 20 min at 4 °C. Following centrifugation, samples of the supernatant were carefully collected for Colony Forming Units (CFU) analysis and aliquoted into prelabeled microtubes to storage at −80 °C until cytokine quantification by ELISA.

The number of viable yeasts in the infected organs was determined by counting the number of CFU, as established by Singer-Vermes et al.^44^ Aliquots of tissue homogenates were plated on brain heart infusion agar containing 4% (v/v) horse serum and 5% Pb192 culture filtrate, incubating at 37 °C and CFU quantified daily until colony numbers remained unchanged. For fungal burden assessment, CFU values were expressed as log_10_ CFU per gram of tissue, thereby normalizing the data to organ weight and minimizing potential bias due to differences in organ size or mass between animals.

For histological analysis, the left lung, liver, and spleen of infected mice were removed and fixed in 10% formalin. Five-micrometer sections were stained with hematoxylin–eosin (for lesion analysis) and silver (for fungal evaluation). Morphometric analysis was performed using a Nikon DXM 1200c camera and Nikon NIS-AR 2.30 software.

### Cytokine Detection

Aliquots of lung, liver, and spleen homogenates, previously stored at −80 °C, were used to measure cytokine levels. After thawing, the samples were added to 96-well plates preincubated with primary antibodies against each cytokine. The assays were performed according to the manufacturer’s recommendations: IL-1β (Cat. No. 887013, Invitrogen), IL-2 (Cat. No. 431001, BioLegend), IL-4 (Cat. No. 431101, BioLegend), IL-6 (Cat. No. 431301, BioLegend), IL-10 (Cat. No. 887105, Invitrogen), IL-12 (Cat. No. 433604, BioLegend), IL-17 (Cat. No. 432501, BioLegend), IL-22 (Cat. No. 887422, Invitrogen), IL-23 (Cat. No. 887230, Invitrogen), MCP-1 (Cat. No. 684295, Invitrogen), GM-CSF (Cat. No. 358048, Invitrogen), TNF-α (Cat. No. 430901, BioLegend), IFN-γ (Cat. No. 887314, Invitrogen), and TGF-β (Cat. No. BMS6084, Invitrogen). Plates were read using a spectrophotometric plate reader (VersaMax, Molecular Devices).

### Assessment of Pulmonary Leukocyte Subpopulations by Flow Cytometry

Lung-infiltrating leukocytes were quantified by multiparametric flow cytometry as previously described by Preite et al.^43^ Following euthanasia, lungs were collected and enzymatically digested in RPMI containing DNase I (0.5 mg/mL) and collagenase IV (1 mg/mL) at 37 °C for 30–40 min. Tissues were homogenized, filtered through 70 μm strainers, and centrifuged at 3,000 × g in complete RPMI supplemented with 3% fetal bovine serum, penicillin/streptomycin, l-glutamine, β-mercaptoethanol, nonessential amino acids, and sodium pyruvate. After centrifugation, red blood cells were lysed, and the remaining cells were washed, counted, and prepared for cytometric analysis.

Total leukocytes (CD45^+^), total myeloid cells (CD45^+^CD11b^+^), dendritic cells (CD45^+^CD11b^+^CD11c^+^F4/80^–^), alveolar macrophages (CD45^+^CD11b^+^F4/80^+^), and neutrophils (CD45^+^CD11b^+^Ly6G^high^) were identified to evaluate whether treatments affected myeloid cell populations. Dendritic cells and macrophages were further characterized for CD40, CD80, CD86, and MHCII expression. CD4^+^ and CD8^+^ T lymphocytes were analyzed, along with activation markers CD25 and CD69. Cell suspensions were adjusted to 1 × 10^6^ cells per well, Fc receptors were blocked with anti-CD16/32 antibodies, and cells were incubated with specific surface antibodies. For functional analysis, CD4+ and CD8+ T cells were stimulated with PMA, ionomycin, and brefeldin A for 6 h to assess intracellular production of IFN-γ (Th1/Tc1), IL-4 (Th2/Tc2), and IL-17 (Th17/Tc17). The transcription factor Foxp3 was evaluated in CD4^+^CD25^+^ regulatory T cells (Treg). After surface staining, cells were fixed, permeabilized, and stained intracellularly according to the manufacturer’s instructions. Samples were fixed in 2% paraformaldehyde and at least 50,000 events were acquired using a FACSLyric flow cytometer (BD Biosciences) using FACSuite software (BD Biosciences). Analysis of surface marker expression was performed using FlowJo software (Tree Star). Antibodies were obtained from BD Biosciences, BioLegend, and BD and were performed according to the manufacturer’s instructions. Flow cytometry data are presented as both frequency (percentage of the indicated population) and absolute number (total cells per lung, calculated by multiplying the frequency by the total viable cell count). Frequency reflects the relative enrichment of a population, while absolute numbers indicate the total cellular load in the organ. These complementary measures provide insight into both the composition and the magnitude of the pulmonary immune response.

### Statistical Analysis

The results were expressed as mean ± standard deviation (SD). Graphing and statistical analysis were performed using GraphPad Prism 9.0 software and were considered after the Student’s *t* test was used to compare the two groups, with *p* < 0.05 considered significant.

## Supplementary Material



## Notes

Gating strategy used in flow cytometry experiments: Selection of viable cells, leukocytes, and myeloid cells; Identification of macrophages, dendritic cells, and neutrophils; Identification of CD4^+^ and CD8^+^ T lymphocytes and their subpopulations of interest (PDF)

## Abbreviations

CFU: colony-forming unit
CsA: cyclosporine A
Mφ: macrophages
PCM: paracoccidioidomycosis
