# Supplementary material for: Pre-Exposure to Cyclosporine A Primes Paracoccidioides brasiliensis to Evade Host Immunity
Source: ACS Infect Dis. 2026 Aug 3;12(8):2727–38. doi: 10.1021/acsinfecdis.6c00285 (PMC13488435; doi:10.1021/acsinfecdis.6c00285)
Supplement: Supplementary file 1 [file id6c00285_si_001.pdf]

SUPPORTING INFORMATION

Pre-exposure to Cyclosporine A Primes Paracoccidioides brasiliensis to evade Host Immunity

Filipe Nogueira Franco[1], Jean de Souza Lisboa Kozlowski[2], Nycolas Willian Preite[1], Coral Molist Homs[1], Luiz Fernando de Oliveira[1], Ana Beatriz Furtado Rodrigues[2], João Henrique Dal Ponte Silva[2], Érica Borges Lima[1], Flávio Vieira Loures[1] and Claudia Barbosa Ladeira de Campos[2]*

[1] Laboratory of Applied Immunology, Institute of Science and Technology, Federal University of São Paulo: Rua Talim, 330 - Vila Nair, São José dos Campos, São Paulo, Brazil, CEP 12231-280.

[2] Laboratory of Biochemistry and Molecular Biology of Fungi (BioFun), Institute of Science and Technology, Federal University of São Paulo: Rua Talim, 330 - Vila Nair, São José dos Campos, São Paulo, Brazil, CEP 12231-280. *E-mail: campos.claudia@unifesp.br

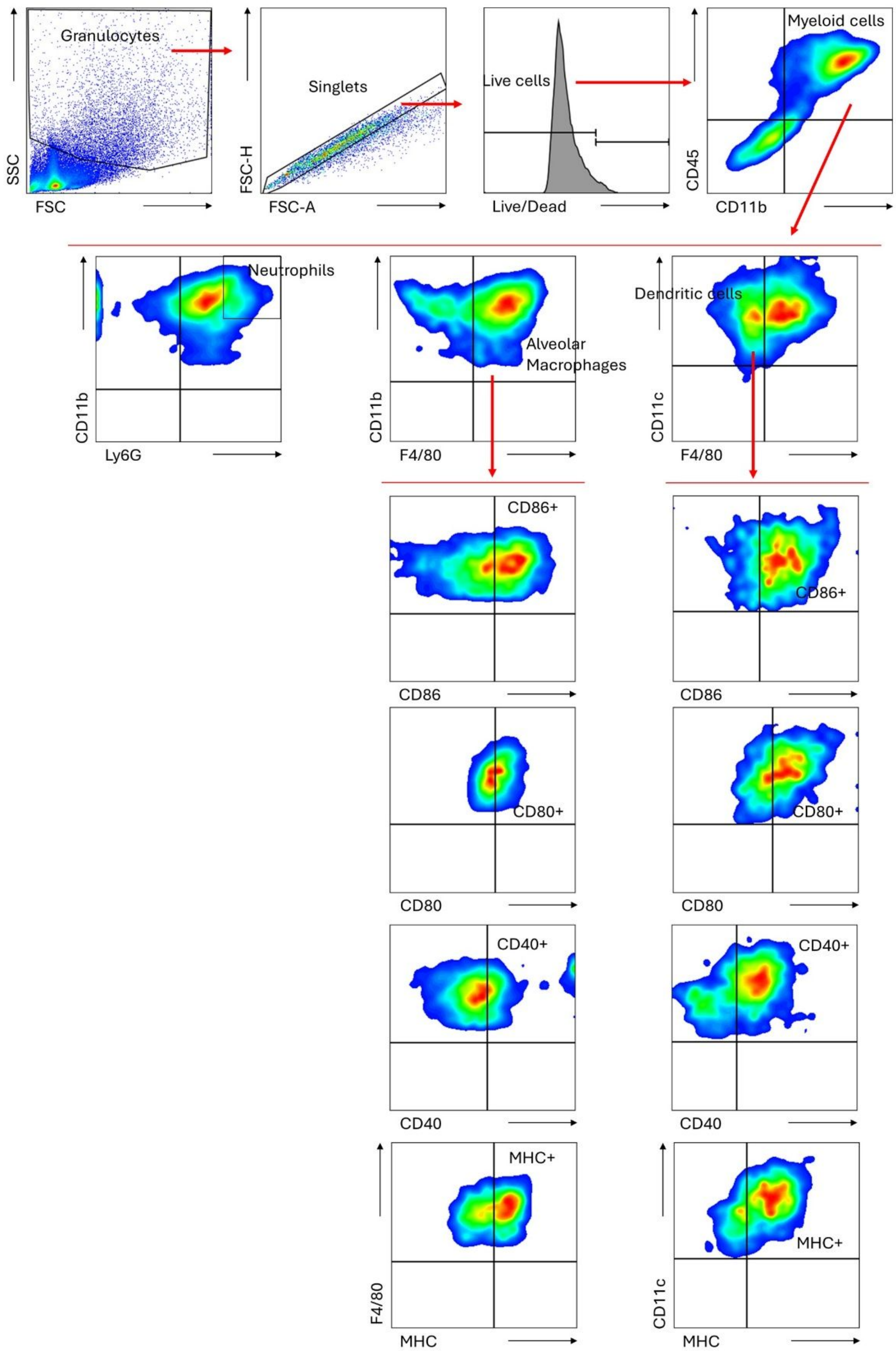
Granulocytes
SSC
FSC
Singlets
FSC-H
FSC-A
Live cells
Live/Dead
Myeloid cells
CD45
CD11b
Neutrophils
CD11b
Ly6G
Alveolar
Macrophages
CD11b
F4/80
Dendritic cells
CD11c
F4/80
CD86+
CD86
CD86+
CD86
CD80+
CD80
CD80+
CD80
CD40+
CD40
CD40+
CD40
MHC+
F4/80
MHC
MHC+
CD11c
MHC

Figure S1. Gating strategy used for the identification of immune cell populations. Myeloid cells from murine tissues were identified by flow cytometry. Cells were initially gated based on forward scatter (FSC) and side scatter (SSC), followed by doublet exclusion and live cell selection. The live single-cell population was then gated for myeloid cells ($CD45^+CD11b^+$). From the myeloid gate, neutrophils ($CD45^+CD11b^+Ly6G^{hi}$), alveolar macrophages ($CD45^+CD11b^+F4/80^+$), and dendritic cells (DCs; $CD45^+CD11b^+CD11c^+F4/80^-$) were identified. Macrophages and DCs were further analyzed for the surface expression of CD80, CD86, CD40, and MHC-II. Spectral compensation was performed using single-stained UltraComp eBeads and single-stained cells for each fluorochrome, and compensation matrices were verified using FMO controls to ensure accurate fluorescence spillover correction. A minimum of 100,000 total events were recorded per sample. Data were acquired on a FACS Lyric flow cytometer (100,000 events per sample) and analyzed using FlowJo software (Tree Star).

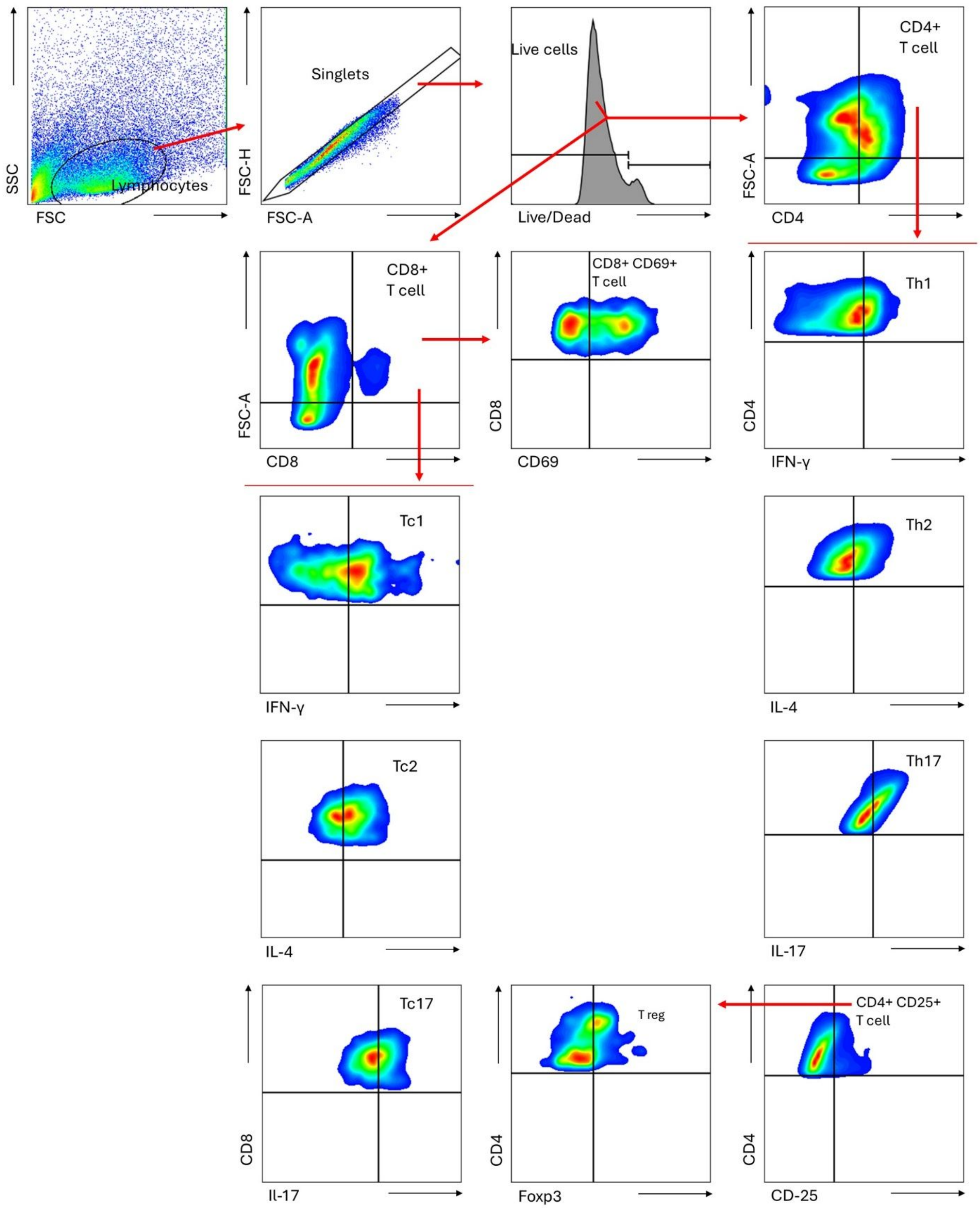

SSC
FSC
Lymphocytes
Singlets
FSC-H
FSC-A
Live cells
Live/Dead
CD4+
T cell
FSC-A
CD4
CD8+
T cell
FSC-A
CD8
CD8+ CD69+
T cell
CD8
CD69
Th1
CD4
IFN-γ
Tc1
IFN-γ
Th2
IL-4
Tc2
IL-4
Th17
IL-17
Tc17
CD8
Il-17
T reg
CD4
Foxp3
CD4+ CD25+
T cell
CD4
CD-25

Figure S2. Gating strategy for the identification of lymphoid populations and T cell subsets. Lymphoid cells from murine tissues were identified by flow cytometry. Cells were initially gated based on forward scatter (FSC) and side scatter (SSC), followed by doublet exclusion and live cell selection. From live single-cell, populations of CD4 T-lymphocytes ($CD4^+$ and $CD4^+CD25^+$), CD8 T-lymphocytes ($CD8^+$ and $CD8^+CD69^+$), Th1 ($CD4^+$IFN-y^+), Th2 ($CD4^+$IL-4^+), Th17 ($CD4^+$IL-17^+), Treg ($CD4^+CD25^+$Foxp3^+), Tc1 ($CD8^+$IFN-y^+), Tc2 ($CD8^+$IL-4^+), and Tc17 ($CD8^+$IL-17^+), were assessed. For intracellular cytokine staining, cells were stimulated with PMA/Ionomycin in the presence of Brefeldin A for 4 hours prior to staining. Spectral compensation was performed using single-stained UltraComp eBeads and single-stained cells for each fluorochrome, and compensation matrices were verified using FMO controls to ensure accurate fluorescence spillover correction. A minimum of 100,000 total events were recorded per sample. Data were acquired on a FACS Lyric flow cytometer (100,000 events per sample) and analyzed using FlowJo software (Tree Star).
